# Targeting the D Series Resolvin Receptor System for the Treatment of Osteoarthritis Pain

**DOI:** 10.1002/art.40001

**Published:** 2017-04-26

**Authors:** Junting Huang, James J. Burston, Li Li, Sadaf Ashraf, Paul I. Mapp, Andrew J. Bennett, Srinivasarao Ravipati, Petros Pousinis, David A. Barrett, Brigitte E. Scammell, Victoria Chapman

**Affiliations:** ^1^ University of Nottingham Nottingham UK

## Abstract

**Objective:**

Pain is a major symptom of osteoarthritis (OA); currently available analgesics either do not provide adequate pain relief or are associated with serious side effects. The aim of this study was to investigate the therapeutic potential of targeting the resolvin receptor system to modify OA pain and pathology.

**Methods:**

Gene expression of 2 resolvin receptors (ALX and ChemR23) was quantified in synovium and medial tibial plateau specimens obtained from patients with OA at the time of joint replacement surgery. Two models of OA joint pain were used for the mechanistic studies. Gene expression in the joint and central nervous system was quantified. The effects of exogenous administration of the D series resolvin precursor 17(R)‐hydroxy‐docosahexaenoic acid (17[R]‐HDoHE) on pain behavior, joint pathology, spinal microglia, and astroglyosis were quantified. Plasma levels of relevant lipids, resolvin D2, 17(R)‐HDoHE, and arachidonic acid, were determined in rats, using liquid chromatography tandem mass spectrometry.

**Results:**

There was a positive correlation between resolvin receptor and interleukin‐6 (IL‐6) expression in human OA synovial and medial tibial plateau tissue. In rats, synovial expression of ALX was positively correlated with expression of IL‐1β, tumor necrosis factor, and cyclooxygenase 2. Treatment with 17(R)‐HDoHE reversed established pain behavior (but not joint pathology) in 2 models of OA pain. This was associated with a significant elevation in the plasma levels of resolvin D2 and a significant reduction in astrogliosis in the spinal cord in the monosodium iodoacetate–induced OA rat model.

**Conclusion:**

Our preclinical data demonstrate the robust analgesic effects of activation of the D series resolvin pathways in 2 different animal models of OA. Our data support a predominant central mechanism of action in clinically relevant models of OA pain.

Osteoarthritis (OA) is a highly prevalent degenerative joint disorder characterized by loss of cartilage, subchondral bone remodeling, and synovial inflammation 1, 2. Pain is the predominant symptom of OA, which limits movement and causes disability 3. OA pain is significantly associated with synovial inflammation and changes in the subchondral bone 4, and evidence of central sensitization and the spread of pain has been reported 5. Existing drugs are poorly effective and/or are associated with adverse side effects. Most often, total joint replacement is the only successful therapeutic treatment 6.

Resolvins are endogenous specialized pro‐resolution lipid mediators derived from docosahexaenoic acid (DHA [D series resolvins]) and eicosapentaenoic acid (EPA [E series resolvins]), which exhibit potent antiinflammatory and pro‐resolution properties 7, 8. Four resolvin receptors have been identified: ALX (also known as *N*‐formyl peptide receptor 2), G protein–coupled receptor 32 (GPR32) 9, 10, 11, chemokine‐like receptor 1 (ChemR23) 12, and leukotriene B_4_ receptor (BLT‐1) 13. Resolvin D1 (RvD1) binds to and activates both ALX and GPR32 in human tissue, while in murine tissue the actions of RvD1 are mediated by ALX 14. ChemR23 and BLT‐1 are the receptors through which RvE1 and RvE2 act 14.

In the context of their therapeutic potential for pain management, RvE1 and RvD1 (administered exogenously) attenuate pain behavior in models of acute inflammatory pain 15, 16 and a model of chronic adjuvant‐induced arthritis 17. RvD1 inhibits the activity of some temperature‐sensitive transient receptor potential (TRP) ion channels expressed by the primary afferent sensory fibers TRP ankyrin 1 (TRPA‐1) 18 and TRP vanilloid channel 3 (TRPV‐3) 19, but not TRPV‐1. Due to the rapid degradation of resolvins, local (intraplantar or intrathecal) routes of administration have been studied. Spinal administration of RvE1 reduced capsaicin‐ and tumor necrosis factor (TNF)–induced spontaneous pain and hypersensitivity in mice and partially attenuated pain behavior in models of neuropathic pain 20. RvD1 and RvE1 can modulate TRPV‐1 and TNF responses in the spinal cord 20, 21, 22 and inhibit phosphorylation of *N*‐methyl‐d‐aspartate receptors and cytokine expression in the spinal cord in the setting of chronic pancreatitis‐induced pain 23. Thus, both peripheral and spinal mechanisms of action contribute to the inhibitory effects of the resolvins in models of inflammatory and neuropathic pain, with predominant peripheral antiinflammatory mechanisms including inhibition of neutrophil infiltration, edema, and proinflammatory cytokine expression 21.

The therapeutic potential of exogenously administered RvD1 and RvE1 may be limited by instability and short durations of action. Treatment with precursors of the active molecules offers an alternative longer‐lasting and beneficial approach 17, as does the development of chemically and metabolically stable analogs such as 17R‐hydroxy‐19‐para‐fluorophenoxy‐resolvin D1 24. Inhibitory effects of a precursor of RvD1, 17(R)‐hydroxy‐docosahexaenoic acid (17[R]‐HDoHE), on mechanical hyperalgesia in a model of inflammatory joint pain have been reported and associated with reductions in hind paw levels of TNF and interleukin‐1β (IL‐1β) and spinal cord expression of NF‐κB and cyclooxygenase 2 (COX‐2) 17. These data suggest that exogenous augmentation of resolvin precursors has therapeutic potential for the treatment of pain states that are underpinned by peripheral and/or central sensitization mechanisms.

The aim of the current study was to provide new clinical and preclinical evidence for the therapeutic potential of the D series resolvin pathway for the treatment of OA pain. Using 2 different clinically relevant models of OA joint pain, we performed mechanistic studies to interrogate the contribution of peripheral joint versus spinal cord sites of action of this novel class of analgesics.

## PATIENTS AND METHODS

### Subjects

Research using clinical samples was approved by generic ethics committees for the Nottingham University Hospitals NHS Trust Biobank (reference no. RSCH 488). Human synovial tissue and bone from the medial tibial plateau were obtained from 15 patients who underwent total knee replacement (TKR) surgery for OA pain. These tissues were selected on the basis of established associations between inflammation, bone remodeling, and pain 1, 2. Fresh tissue samples were collected from the surgical team and snap‐frozen and stored in a −80ºC freezer at the Biobank, City Hospital, University of Nottingham. Fresh synovial tissue specimens (n = 15) and medial tibial plateau specimens (n = 14) were used to quantify gene expression.

### Animals and model induction

Animal experiments were approved by the Nottingham University ethics committee, and all procedures were approved by the UK Home Office in accordance with the Animals (Scientific Procedures) Act 1986 and conform to the guidelines of the International Association for the Study of Pain. Adult male Sprague‐Dawley rats (n = 166) were used (Charles River). All procedures and testing were performed in a blinded manner. The model of monosodium iodoacetate (MIA)–induced OA pain was generated as previously described 25. The medial meniscal transection (MNX) induction model of OA pain was based on previously described methods 26 (see also Supplementary Methods, available on the *Arthritis & Rheumatology* web site at http://onlinelibrary.wiley.com/doi/10.1002/art.40001/abstract).

### Pharmacologic interventions and assessment of pain behavior

Weight‐bearing asymmetry and hind paw mechanical withdrawal thresholds were determined using a Linton incapacitance tester and von Frey monofilaments (Linton Instrumentation; bending force 1–26*g*, respectively) as previously described 27 (see Supplementary Methods). An RvD1 precursor, 17(R)‐HDoHE, also known as 17(R)‐HDHA, was purchased from Cayman Chemical. This precursor gives rise to the production of 17R RvD1 and 17R RvD2, epimers of endogenous RvD1, RvD2, RvD3, and RvD4 (synthesized from 17[S]‐HDoHE), and aspirin‐triggered epimers (17R forms), when COX‐2 is acetylated 14.

The 17(R)‐HDoHE (stock solution 100 μg/ml in ethanol) was diluted in normal sterile saline to provide a concentration of 1 ng/μl. The vehicle solution consisted of 1% ethanol solution in 99% saline. A series of different pharmacologic studies were performed using 300 ng of 17(R)‐HDoHE in 300 μl saline administered intraperitoneally. Study 1 determined the effects of a single injection of 17(R)‐HDoHE on pain behavior on day 14 after induction of the model. Study 2 determined the effects of repeated administration of 17(R)‐HDoHE (300 ng in 300 μl saline, every other day from day 14 until day 28 after induction of the model) on pain behavior in the MIA‐induced OA pain model and the MNX induction model of OA pain. Study 3 determined the effects of discontinuous administration of 17(R)‐HDoHE (300 ng in 300 μl saline, from day 14 to day 22 after induction of the model) on pain behavior, quantified until day 35 after induction of the model. All of the drug intervention studies were conducted in a blinded manner.

### Quantitative real‐time polymerase chain reaction

At the end of the behavioral studies, the rats were killed via an overdose of sodium pentobarbital, and fresh‐frozen spinal cords and synovial tissue were collected and stored at −80ºC. Tissues were homogenized in ice‐cold TRI Reagent to extract total RNA from the samples, as previously described 26. Human OA synovial tissue collected at the time of TKR surgery was homogenized in cold TRI Reagent, and RNA was extracted as previously described. Bone from the mid part of the medial tibial plateau collected at the time of TKR surgery was pulverized in liquid nitrogen, and RNA was extracted in TRI Reagent. RNA samples were kept in a −80ºC freezer for future use (see Supplementary Methods, available on the *Arthritis & Rheumatology* web site at http://onlinelibrary.wiley.com/doi/10.1002/art.40001/abstract). Expression of target genes was quantified using previously described methods 26, 28. Primers and probes were designed using Primer Express 3.0 software (Applied Biosystems) and synthesized by personnel at MWG Biotech, and minor groove binder probes were biosynthesized by personnel at Applied Biosystems (see Supplementary Methods).

### Glial cell immunofluorescence analysis

Rats were killed by sodium pentobarbital overdose and transcardially perfused with saline and then 4% paraformaldehyde, pH 7.4 (Sigma). The lumbar spinal cord was removed, postfixed in 4% paraformaldehyde, and stored in 30% sucrose. The spinal cord was then sectioned, and immunohistochemical analysis was performed using mouse anti–glial fibrillary acidic protein (anti‐GFAP) antibodies (1:100) (Fisher Scientific), as previously described 26 (see Supplementary Methods).

### Histologic staining and scoring of knee joints

Cartilage histopathology was scored from 0 (normal) to 5 (severe degeneration), and a total joint damage score (range 0–15) was obtained by combining the cartilage score with the score for joint involvement (range 0–3) 29. Synovial inflammation was graded on a scale of 0 (lining layer, 1–2 cells thick) to 3 (lining layer >9 cells thick and/or severe increase in cellularity), as previously described 29. Sections from the posterior half of the knee joints were dewaxed and recalcified with calcium chloride and magnesium chloride before tartrate‐resistant acid phosphatase (TRAP) staining was conducted using a commercially available kit (F386A; Sigma‐Aldrich). TRAP‐positive osteoclasts were quantified as previously described 29 (see also Supplementary Methods, available on the *Arthritis & Rheumatology* web site at http://onlinelibrary.wiley.com/doi/10.1002/art.40001/abstract).

### Statistical analysis

Data were analyzed with GraphPad Prism version 5 or 6 and are presented as the mean ± SEM. Behavioral data were analyzed by two‐way analysis of variance (ANOVA) with Bonferroni post hoc correction. Histologic scoring was analyzed by one‐way ANOVA with Bonferroni post hoc test or by Kruskal‐Wallis ANOVA followed by Dunn's post hoc test for nonparametric data. Gene expression levels were analyzed by unpaired *t*‐test (parametric data) or Mann‐Whitney test (nonparametric data). Correlations between gene expression of resolvin receptors and pain behavior or genes of interest were analyzed using Pearson's correlation coefficient (parametric data) or Spearman's correlation coefficient (nonparametric data) analysis. For immunofluorescence analysis, data were analyzed by one‐way ANOVA with Bonferroni post hoc test. Correlations between spinal GFAP expression and weight‐bearing asymmetry and the ipsilateral paw withdrawal threshold were determined by a Spearman's correlation.

## RESULTS

### Expression of ChemR23 and ALX messenger RNA (mRNA) in the OA joint

**Figure 1:**
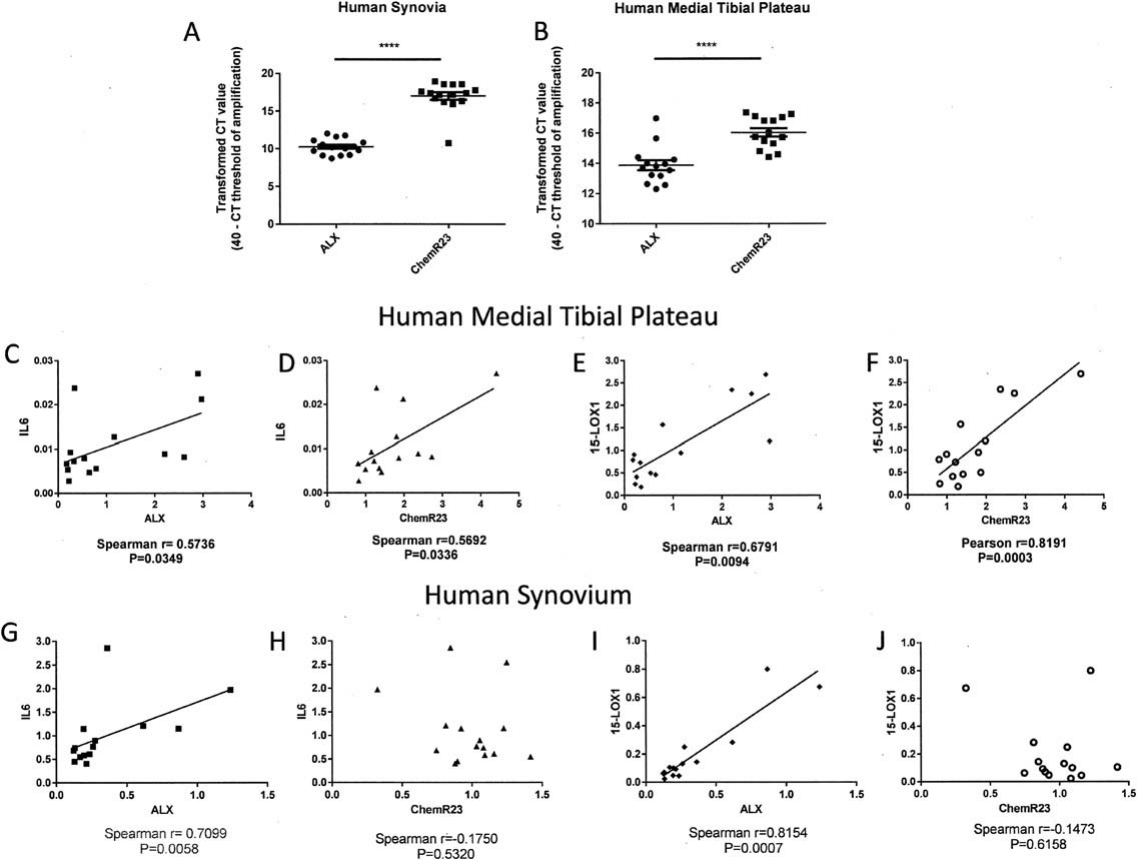
Expression of resolvin receptors ALX and ChemR23 in human osteoarthritis (OA) joint tissue. **A** and **B,** Transformed C_t_ values for resolvin receptors ALX and ChemR23 mRNA in synovium (**A**) and medial tibial plateau (**B**) specimens obtained from patients with end‐stage OA. Expression of ChemR23 was significantly higher than that of ALX in both OA synovium (n = 15 specimens) (**A**) and medial tibial plateau (n = 14 specimens) (**B**), as shown by a larger transformed C_t_ value (maximum cycle number for run – cycle number at which exponential amplification occurs). Bars show the mean ± SEM. ∗∗∗∗ = *P* < 0.0001 by unpaired *t*‐test. **C–F,** Correlations of ALX and ChemR23 with the cytokine interleukin‐6 (IL‐6) and the enzyme 15‐lipoxygenase 1 (15‐LOX‐1) in human medial tibial plateau tissue. **G–J,** Correlations of ALX and ChemR23 with IL‐6 and 15‐LOX‐1 in human synovium.

Both ALX and ChemR23 were present in human synovium and medial tibial plateau bone obtained following TKR surgery for OA (Figures 1A and B). For the synovium, there was an approximate 6‐C_t_ difference between ChemR23 and ALX, and for the medial tibial plateau bone there was a 2‐C_t_ difference, indicating higher expression of ChemR23 compared with ALX in both tissues. Given the role of these receptors in regulating inflammatory signaling, it is noteworthy that the expression of both ChemR23 and ALX was positively correlated with mRNA expression of IL‐6 in the medial tibial plateau bone (Figures 1C and D). Expression of both ChemR23 and ALX was also positively correlated with expression of the enzyme 15‐lipoxygenase 1 (15‐LOX‐1) in the medial tibial plateau bone (Figures 1E and F). In the synovium, correlations were less robust. Expression of ALX but not ChemR23 was positively correlated with IL‐6 expression (Figures 1G and H), and expression of ALX was also positively correlated with 15‐LOX‐1 expression (Figures 1I and J). There was no correlation between body mass index (BMI) or age with expression of ALX, ChemR23, or any other genes studied in the synovium of OA patients (data not shown). Analysis of the correlation of BMI and age with expression of selected genes in medial tibial plateau bone from OA patients revealed a significant negative correlation between BMI and the expression of TNF. There was a significant negative correlation between age and expression of ChemR23 and 15‐LOX‐1 (data not shown).

**Figure 2:**
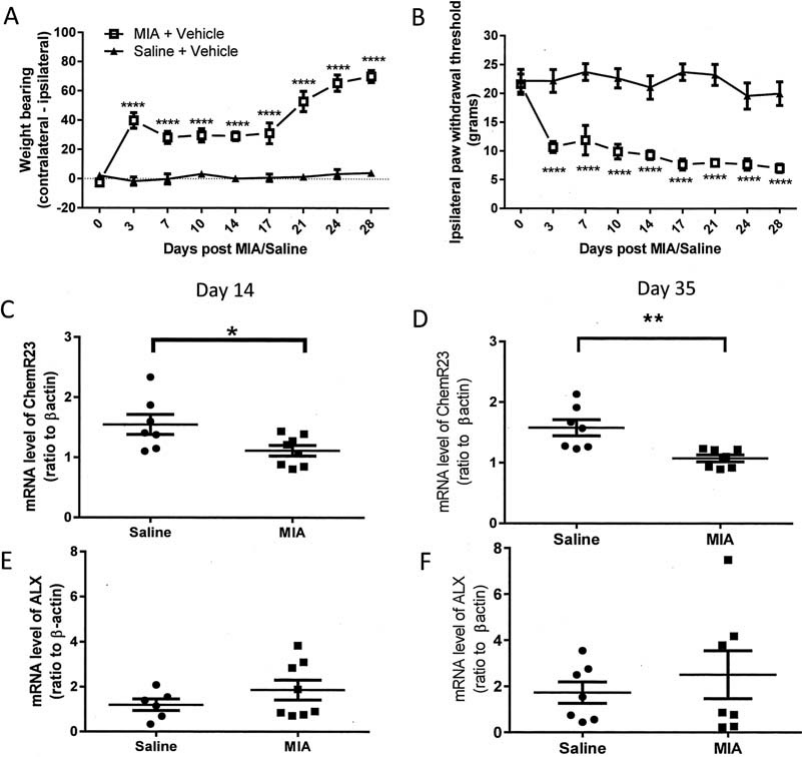
Expression of resolvin receptors ALX and ChemR23 in rat osteoarthritis (OA) joint tissue. **A** and **B,** Significant weight‐bearing asymmetry (**A**) and decreased ipsilateral paw withdrawal thresholds (**B**) following intraarticular injection of monosodium iodoacetate (MIA) into the knee joints (MIA‐treated rats) compared with saline‐treated control rats (n = 8 per group). Values are the mean ± SEM. ∗∗∗∗ = *P* < 0.0001 versus control, by two‐way analysis of variance with Bonferroni's post hoc test. **C** and **D,** Decreased synovial expression of ChemR23 in MIA‐treated rats compared with saline‐treated control rats on day 14 (**C**) and day 35 (**D**). **E** and **F,** Comparable expression of ALX in the synovium of MIA‐treated rats and saline‐treated rats on day 14 (**E**) and day 35 (**F**) (n = 7–8 per group). Bars in **C**–**F** show the mean ± SEM. ∗ = *P* < 0.05; ∗∗ = *P* < 0.01 by unpaired *t*‐test.

The preclinical MIA model of OA pain was associated with marked weight‐bearing asymmetry (*P* < 0.0001 versus saline + vehicle) (Figure 2A). In addition, MIA‐induced OA pain was associated with reductions in ipsilateral paw withdrawal thresholds (*P* < 0.0001 versus saline + vehicle) (Figure 2B), as previously described 25. Consistent with the clinical data, synovium from saline‐treated (control) rats expressed both ChemR23 and ALX (Figures 2C–F). There was a significant reduction in ChemR23 expression in the synovium at both the earlier (day 14) (Figure 2C) and later (day 35) (Figure 2D) time points in MIA‐treated animals compared with saline‐treated controls. Synovial expression of ALX was unaltered in MIA‐injected rats compared to saline‐treated rats at either time point studied (Figures 2E and F).

**Figure 3:**
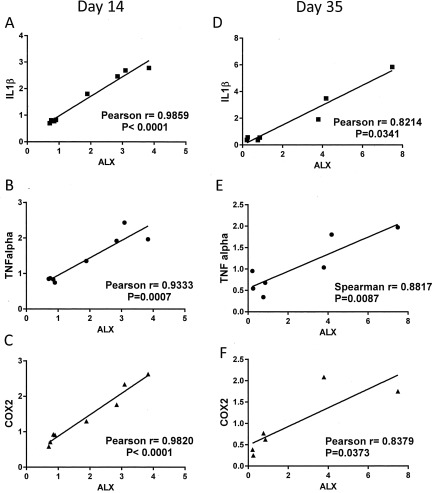
Correlations between synovial expression of ALX and markers of inflammation in rat synovium. Significant correlations between ALX expression and synovial expression of interleukin‐1β (IL‐1β), tumor necrosis factor (TNF), and cyclooxygenase 2 (COX‐2) on day 14 (**A**–**C**) and day 35 (**D**–**F**) in monosodium iodoacetate–treated rats (n = 7) were found.

Consistent with the clinical data, there was a trend toward a correlation between ALX and IL‐6 expression (results not shown) at 14 days after induction of the model (r = 0.6826, *P* = 0.0621). At this time point, synovial ALX expression was correlated with IL‐1β, TNF, and COX‐2 expression (Figures 3A–C). At the later time point, synovial ALX expression was correlated with IL‐1β, TNF, and COX‐2 expression in the synovium (Figures 3D–F). There were no significant correlations between ALX expression and IL‐6, IL‐1β, TNF, and COX‐2 expression in the synovium of control (saline‐treated) rats (data not shown). There were no significant correlations between synovial expression of ChemR23 and IL‐1β, TNF, and COX‐2 in the MIA‐induced model of OA at either time point (data not shown).

The number of ALX‐positive and ChemR23‐positive cells in the synovium was compared in the MIA‐treated rats (day 28 after the MIA injection) and saline‐treated controls. The number of DAPI‐positive nuclei, ED1/CD68‐positive cells, and the number of ALX‐positive and ChemR23‐positive cells in synovial sections was evaluated. The number of DAPI‐positive cells was increased (*P* < 0.01) in the synovium of rats with MIA‐induced OA compared with saline‐injected control rats (see Supplementary Figures 1A and B, available on the *Arthritis & Rheumatology* web site at http://onlinelibrary.wiley.com/doi/10.1002/art.40001/abstract). In addition, the number of ED1‐positive cells (see Supplementary Figures 1A and C) was also increased in the rats with MIA‐induced OA (*P* < 0.005), which is indicative of the likely infiltration of ED1‐positive macrophages in this model. Despite the increase in the number of macrophages in the synovium of rats with MIA‐induced OA pain, the number of ALX‐positive and ChemR23‐positive cells in the synovium was significantly reduced in MIA‐treated rats compared with saline‐treated rats (see Supplementary Figures 1A, D, and E) (*P* < 0.05 for both ALX and ChemR23).

In order to confirm that the antibody staining was not attributable to autofluorescence, we conducted negative control experiments with omission of the primary antibodies (see Supplementary Figure 2, available on the *Arthritis & Rheumatology* web site at http://onlinelibrary.wiley.com/doi/10.1002/art.40001/abstract), in which positively labeled cells were not evident either visually or by velocity analysis. We attempted colocalization experiments for ED1‐positive cells and ALX and ChemR23, but unfortunately we were unable to obtain sufficient quality of staining when these antibodies were applied to synovial sections for analysis.

### Reversal of MIA‐ and MNX‐induced OA pain by the D series precursor 17(R)‐HDoHE

**Figure 4:**
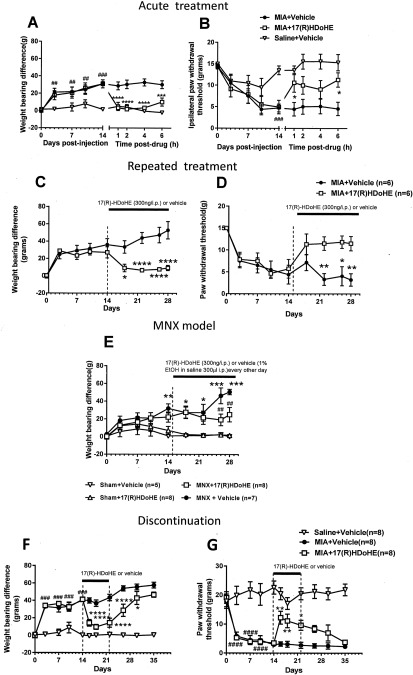
Inhibition of pain behavior by the D series resolvin precursor 17(R)‐hydroxy‐docosahexaenoic acid (17[R]‐HDoHE). **A** and **B,** Attenuated weight‐bearing asymmetry (**A**) and decreased ipsilateral hind paw withdrawal thresholds (**B**) 14 days after model induction in rats treated with monosodium iodoacetate (MIA) + 17(R)‐HDoHE compared to MIA‐injected vehicle‐treated rats. Bars show the mean ± SEM (n = 8 per group). ## = *P* < 0.01; ### = *P* < 0.001 versus saline + vehicle. ∗ = *P* < 0.05; ∗∗∗ = *P* < 0.001; ∗∗∗∗ = *P* < 0.0001 versus MIA + vehicle, by two‐way analysis of variance (ANOVA) with Bonferroni's post hoc test. **C** and **D,** Sustained inhibition of weight‐bearing asymmetry (**C**) and decreased paw withdrawal thresholds (**D**) from day 14 to day 28 following repeated administration of 17(R)‐HDoHE in rats treated with MIA compared with rats treated with MIA+ vehicle. Bars show the mean ± SEM. ∗ = *P* < 0.05; ∗∗ = *P* < 0.01; ∗∗∗∗ = *P* < 0.0001 versus MIA + vehicle, by two‐way ANOVA with Bonferroni's post hoc test. **E,** Attenuated weight‐bearing asymmetry in rats with medial meniscal transection (MNX)–induced osteoarthritis pain treated with repeated administration of 17(R)‐HDoHE compared with those treated with vehicle. Bars show mean ± SEM. ∗ = *P* < 0.05; ∗∗ = *P* = 0.01; ∗∗∗ = *P* < 0.001 versus sham + vehicle. ## = *P* < 0.01 versus MNX + vehicle, by two‐way ANOVA with Bonferroni's post hoc test. **F** and **G,** Gradual return of weight‐bearing asymmetry and decreased hind paw withdrawal thresholds within 7 days following cessation of 7‐day treatment with 17(R)‐HDoHE. Bars show the mean ± SEM. ### = *P* < 0.001; #### = *P* < 0.0001 versus saline + vehicle. ∗ = *P* < 0.05; ∗∗ = *P* < 0.01; ∗∗∗∗ = *P* < 0.0001 versus MIA + vehicle, by two‐way ANOVA with Bonferroni's post hoc test. Values are the mean ± SEM. EtOH = ethanol.

In a series of intervention studies, we evaluated the ability of systemic administration of 17(R)‐HDoHE to reverse pain behavior in 2 models of OA. Systemic administration produced a pronounced and complete reversal of MIA‐induced weight‐bearing asymmetry and restored ipsilateral paw withdrawal thresholds toward control values at 1 hour after administration; these effects lasted for 6 hours (Figures 4A and B). Importantly, the inhibitory effects of 17(R)‐HDoHE on both weight‐bearing asymmetry and hind paw withdrawal thresholds were sustained following repeated administration of 17(R)‐HDoHE for 14 days (Figures 4C and D); there was no evidence of tolerance to this analgesic effect. To consolidate the evidence that these inhibitory effects of the resolvin precursor on MIA‐induced pain behavior has translational relevance, the effects of 17(R)‐HDoHE on pain behavior were also evaluated in the MNX‐induced model of OA. In this analysis, systemic administration of 17(R)‐HDoHE (300 ng intraperitoneally every other day from day 14 after model induction) significantly halted further increases in MNX‐induced weight‐bearing asymmetry (Figure 4E).

The final series of pharmacologic experiments determined the extent to which 17(R)‐HDoHE administration altered pain behavior once treatment had ceased. Following a 7‐day treatment protocol with 17(R)‐HDoHE (days 14–22 after MIA/saline injection), pain behavior was assessed for a further 13 days. It was evident that the analgesic effects of 17(R)‐HDoHE were sustained over a short period of time once treatment had ceased, and then pain behavior returned to levels observed in saline‐treated rats with MIA‐induced OA pain (Figures 4F and G).

**Figure 5:**
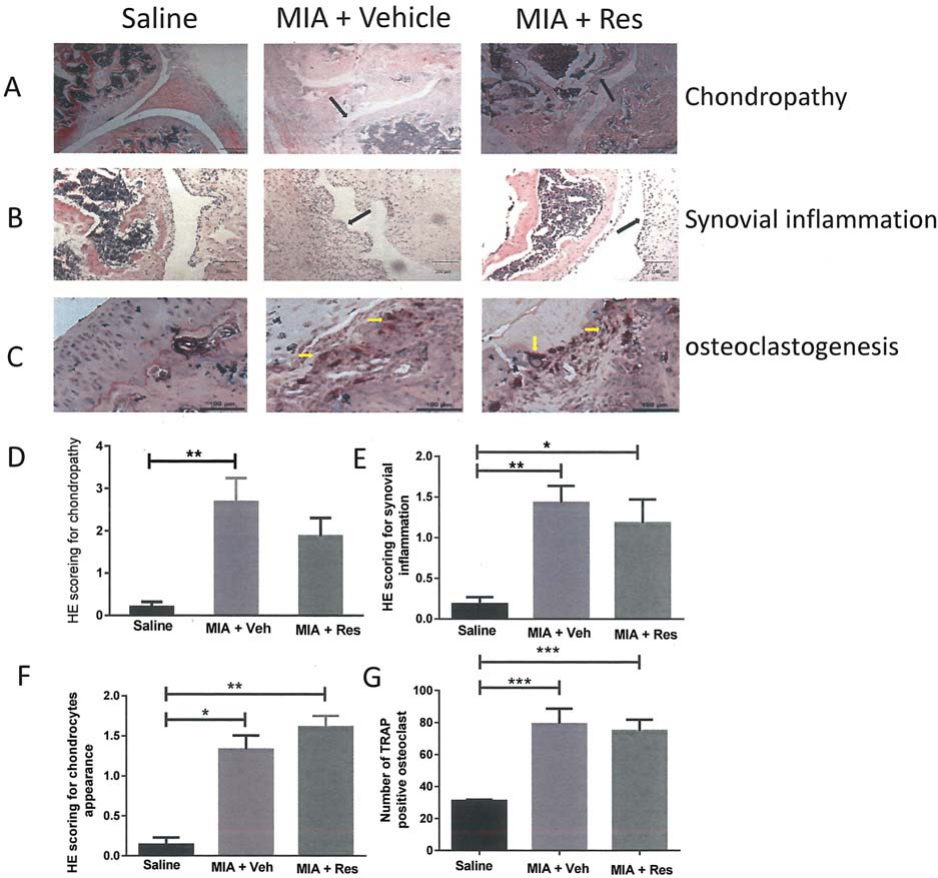
No alteration of osteoarthritis joint pathology by 17(R)‐HDoHE (Res). **A–C,** Representative images of hematoxylin and eosin (H&E)–stained knee joint sections from rats treated with saline, MIA + vehicle (Veh), or MIA + 17(R)‐HDoHE. A, Chondropathy. Bars = 500 μm. **B,** Synovial inflammation. Bars = 100 μm. **C,** Osteoclastogenesis. Bars = 100 μm. **Black arrows** indicate areas of chondropathy and synovial inflammation; **yellow arrows** indicate osteoclastogenesis. **D–G,** Significant joint pathology at 28 days in rats treated with intraarticular injections of MIA compared with saline‐treated rats. Repeated administration of 17(R)‐HDoHE from day 14 to day 28 did not alter the chondropathy score (**D**), synovial inflammation (**E**), chondrocyte appearance (**F**), or number of tartrate‐resistant acid phosphatase (TRAP)–positive osteoclasts (**G**) in the MIA‐treated rats. Bars show the mean ± SEM. ∗ = *P* < 0.05; ∗∗ = *P* < 0.01; ∗∗∗ = *P* < 0.001 by one‐way ANOVA with Bonferroni's post hoc test (parametric data) or Kruskal‐Wallis test with Dunn's post hoc test (nonparametric data). See Figure 4 for other definitions.

To further investigate the potential mechanisms underlying the effects of17(R)‐HDoHE on OA‐induced pain behavior, the effects of repeated treatment with 17(R)‐HDoHE on joint pathology were determined (Figures 5A–C). Intraarticular injection of MIA was associated with a significant increase in chondropathy, synovitis, and chondrocyte appearance and increased numbers of subchondral osteoclasts (Figures 5D–G). Following repeated administration of 17(R)‐HDoHE (300 ng in 300 μl every other day from day 14 to day 28) there were no significant changes in any of these features of OA joint pathology (Figures 5D–G). Similarly, repeated treatment with 17(R)‐HDoHE did not alter MNX‐induced joint pathology (data not shown).

### Spinal effects of 17(R)‐HDoHE correlated with behavioral analgesia

**Figure 6:**
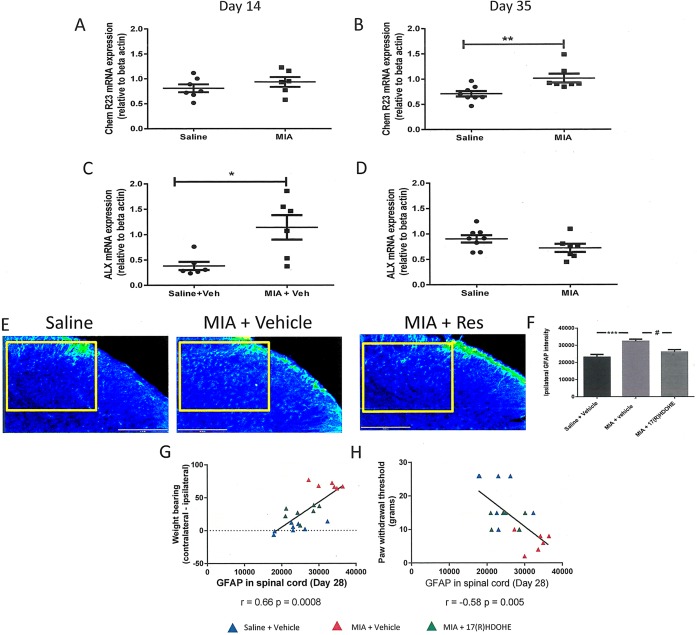
Expression of the resolvin receptors in the spinal cord. **A** and **B,** ChemR23 expression in the ipsilateral dorsal horn of the lumbar spinal cord (L3–L5) on day 14 (**A**) and day 35 (**B**) in MIA‐treated rats compared with saline‐treated rats. **C** and **D,** ALX expression in the ipsilateral dorsal horn of the lumbar spinal cord (L3–L5) on day 14 (**C**) and day 35 (**D**) in MIA‐treated rats compared with saline‐treated rats. Bars show the mean ± SEM (n = 5–6 per group). ∗ = *P* < 0.05; ∗∗ = *P* < 0.01 by unpaired *t*‐test. **E,** Anti–glial fibrillary acidic protein (GFAP) immunofluorescence in the ipsilateral L4 dorsal horn of the spinal cord 28 days following induction of the MIA model of OA pain, indicative of increased astrocyte reactivity. Repeated systemic treatment with 17(R)‐HDoHE (300 ng every other day from day 14 to day 28 after model induction) resulted in a significant decrease in MIA‐induced GFAP immunofluorescence. The boxed area shows the area evaluated for GFAP quantification. **F,** Quantification of GFAP fluorescence. Bars show the mean ± SEM (n = 7–8 rats per group). ∗∗∗ = *P* < 0.001; # = *P* < 0.05 by one‐way ANOVA. **G,** Positive correlation between GFAP expression in the ipsilateral dorsal horn of the spinal cord and weight‐bearing asymmetry in all of the treatment groups. **H,** Negative correlation between GFAP expression in the ipsilateral dorsal horn of the spinal cord and the paw withdrawal threshold in all of the treatment groups. See Figure 4 for other definitions.

Given the lack of effect of 17(R)‐HDoHE on joint pathology in 2 models of OA pain, we investigated potential spinal mechanisms underlying these effects. ChemR23 expression in the spinal cord on day 14 in MIA‐treated rats was comparable with that in saline‐treated controls (Figure 6A); however, expression was increased on day 35 in MIA‐treated rats compared with saline‐treated controls (Figure 6B). The expression of ALX in the ipsilateral dorsal horn of the spinal cord was increased in MIA‐treated rats compared with saline‐treated rats on day 14 (Figure 6C), while on day 35 there was no difference in spinal ALX expression between MIA‐treated and saline‐treated rats (Figure 6D).

We previously reported a significant increase in GFAP immunofluorescence, a marker for astrogliosis, in the spinal cord at later time points in the MIA‐induced model of OA 25. Consistent with previous findings, GFAP immunofluorescence was significantly increased in the ipsilateral dorsal horn (Figures 6E and F) but not the contralateral dorsal horn (data not shown) of MIA‐treated rats that received vehicle, compared with saline‐treated controls. Repeated systemic administration of 17(R)‐HDoHE (300 ng in 300 μl every other day from day 14 to day 28) significantly inhibited GFAP immunofluorescence in the ipsilateral dorsal horn of the spinal cord in MIA‐treated rats, compared with vehicle‐treated rats with MIA‐induced OA pain (Figures 6E and F). Correlation analysis revealed that spinal GFAP expression was positively correlated with weight‐bearing asymmetry (Figure 6G) and negatively correlated with ipsilateral paw withdrawal thresholds (Figure 6H).

### Liquid chromatography tandem mass spectrometry (LC‐MS/MS) quantitative analysis of resolvins and 17(R)‐HdoHE

Plasma levels of 45 oxylipins, including arachidonic acid, RvD1, RvD2, and the precursor 17(R)‐HDoHE were quantified 150 minutes following systemic administration of 17(R)‐HDoHE in MIA‐treated rats, vehicle‐treated rats, and saline‐treated controls. Levels of arachidonic acid were not altered by systemic administration of 17(R)‐HDoHE (see Supplementary Figures 3A and D, available on the *Arthritis & Rheumatology* web site at http://onlinelibrary.wiley.com/doi/10.1002/art.40001/abstract). As expected at the time point studied, plasma levels of 17(R)‐HDoHE were not altered following 17(R)‐HDoHE pretreatment (see Supplementary Figures 3B and E), but levels of RvD2 were significantly increased in 17(R)‐HDoHE–treated rats with MIA‐induced OA compared with vehicle‐treated rats with MIA‐induced OA (see Supplementary Figures 3C and F). Although there were no significant differences in the group data for plasma levels of RvD1, this lipid was detected in a larger number of samples following 17(R)‐HDoHE treatment (5 of 8 samples) compared with those that received vehicle (3 of 8). Of the remaining oxylipins quantified, only 9‐oxo‐10E,12Z‐octadecadienoic acid (9‐oxoODE) and 13‐oxoODE were significantly increased in the group of rats with MIA‐induced OA treated with 17(R)‐HDoHE compared with the group that received saline plus vehicle (data not shown).

## DISCUSSION

Herein we report that the resolvin receptors ALX and ChemR23 are expressed at the mRNA level in both the synovium and tibial plateau of OA patients. ALX expression was positively correlated with expression of IL‐6, which is a clinically relevant knee pain biomarker in patients with early OA and those with advanced‐stage knee OA 30, and positively correlated with expression of 15‐LOX‐1, a key enzyme involved in D series resolvin generation, in both OA synovium and medial tibial plateau bone. Associations between ChemR23, 15‐LOX‐1, and IL‐6 expression were less consistent for both tissues. The enzyme 15‐LOX‐1 is involved in the biosynthesis of D series resolvins from DHA but not the production of E series resolvins from EPA; therefore, correlations between 15‐LOX‐1 and ChemR23 may reflect the more general role of 15‐LOX‐1 in inflammatory pathways. Although correlations between these inflammatory mediators and resolvin receptor expression do not necessarily reflect a causal relationship, they do support the need for further investigation of the role of the resolvin system in OA mechanisms. Due to the lack of availability of fresh non‐OA knee synovium and tibial plateau bone, we were unable to evaluate whether expression of ALX and ChemR23 is altered in end‐stage OA. To overcome this inevitable hurdle of clinical research, preclinical studies using well‐established models of OA pain in the rodent were undertaken.

Both ALX and ChemR23 were present in control rat synovium at the early and late time points in the model of MIA‐induced OA pain. The impact of this model of OA on the expression of these receptors differed. Expression of ChemR23 mRNA was reduced in the synovium at both the early and late time points in the MIA model of OA. In contrast, synovial expression of ALX mRNA in MIA‐injected rats remained stable and comparable with levels in saline‐treated rats at both time points in the model of MIA‐induced OA pain. Synovial expression of ALX mRNA in the MIA‐induced model of OA pain was positively correlated with key inflammatory genes (TNF, IL‐1β, IL‐6, and COX‐2), which is consistent with the presence of synovial inflammation in this model of OA pain, described herein and in previous studies 31, 32, 33, and the expression of ALX by neutrophils, macrophages, and fibroblast‐like synoviocytes 34.

In addition to the findings of the gene expression studies, we also demonstrated an increased number of ED1‐positive cells in the synovium of rats with MIA‐induced pain compared with control rats, reflecting a likely increase in macrophage infiltration. These events were associated with a decrease in the number of ChemR23‐positive and ALX‐positive cells in the synovium of MIA‐treated rats compared with controls. Thus, there was a consistent direction of effect for ChemR23 mRNA and protein. In contrast, ALX mRNA expression was not altered, but the numbers of ALX‐positive cells were decreased in the synovium of MIA‐treated rats, which may reflect posttranslational changes in this receptor.

Systemic administration of the D series resolvin precursor 17(R)‐HDoHE produced robust inhibition of established pain behavior in both the chemically induced and surgically induced OA pain models. Systemic treatment with a single dose of 17(R)‐HDoHE rapidly reversed established weight‐bearing asymmetry, and this reversal was evident 1 hour posttreatment and was sustained for 6 hours. LC‐MS/MS analysis of plasma confirmed that this treatment significantly increased plasma levels of RvD2 and increased the number of samples in which RvD1 was detectable. The dose of 17(R)‐HDoHE studied was based on the comprehensive pharmacologic evaluation of 17(R)‐HDoHE in a model of inflammatory arthritis 17. Consistent with this previous study, we observed that a very low dose of 17(R)‐HDoHE has beneficial effects on pain behavior, and that repeated treatment with 17(R)‐HDoHE has a sustained inhibitory effect on pain behavior over a 2‐week period in both the chemically induced and surgically induced models of OA pain. Although there were subtle differences in the rapidity of onset and magnitude of the inhibitory effects of 17(R)‐HDoHE between the 2 models of OA pain, overall this treatment had a comparable inhibitory effect in the 2 models. Unlike opioid‐based analgesics, sustained treatment with 17(R)‐HDoHE did not lead to tolerance.

To further investigate the underlying mechanisms leading to the beneficial effects of 17(R)‐HDoHE, the effects of repeated treatment on joint pathology were quantified in the model of MIA‐induced pain. Consistent with previous studies 26, 35, 36, 37 and the key clinical features of OA, the model of MIA‐induced OA was associated with significant cartilage damage, synovial inflammation, and increased numbers of subchondral osteoclasts. Despite the robust analgesic effects of 17(R)‐HDoHE, this treatment did not alter any of the features of knee joint pathology. This was also the case in the model of MNX‐induced OA pain and joint pathology. This observation is consistent with our demonstration that the numbers of ALX‐positive cells were reduced in the synovium of rats with MIA‐induced OA pain compared with saline‐treated controls, which is likely to limit/reduce any possible effects of 17(R)‐HDoHE at this level. We previously showed that treatments that act to reduce osteoclast function can alter the progression of joint pathology under identical experimental conditions 29. Unlike RvE1, which can inhibit osteoclasts and bone resorption 38 and protects against bone loss 39, 40, evidence for a role of RvD1 in bone modulation is sparse. It is feasible, however, that a higher dose of 17(R)‐HDoHE may alter pathologic knee changes seen in these models of OA. Overall, our in vivo data demonstrate that 17(R)‐HDoHE can robustly block pain behavior in the MIA model of OA in the face of overt joint damage and synovial inflammation.

Once treatment with 17(R)‐HDoHE was stopped, pain behavior was blocked for an additional 5–7 days, suggesting that augmentation of the resolvin system has longer‐term inhibitory effects on nociceptive signaling, which may represent alterations in both channel activity and signaling pathways. Chronic pain states are often associated with changes in the spinal signaling pathways and increased excitability of spinal neurones, coupled with increased activation of proinflammatory signaling pathways and changes in the activation state of microglia and astrocytes 41.

RvD1 is known to suppress TRPA‐1, TRPV‐3, and TRPV‐4 channel activity in primary sensory fibers 18, 21; therefore, systemic administration of 17(R)‐HDoHE may act to reduce sensory nerve activity arising from the damaged knee joint. It is noteworthy that deletion of TRPA‐1 attenuates joint pathology and pain behavior in the mouse model of MIA‐induced OA 42. It is possible that 17(R)‐HDoHE may still have effects at the level of the joint by activating ALX receptors on synovial cells and possibly reducing release of synovium‐derived nerve sensitization factors such as NGF 43.

Direct spinal administration of RvD1 inhibited evoked pain behavior in models of acute and chronic pain 18, 21, 22; similarly, spinal administration of 17(R)‐HDoHE attenuated inflammation‐induced mechanical hypersensitivity 22. Although the spinal mechanisms underlying the effects of RvD1 are not fully established, common pathways implicated include reductions in TNF release 22 and inhibition of ERK signaling 21. In the current study, immunohistochemical analysis revealed that both ChemR23 and ALX expression in the ipsilateral dorsal horn of the spinal cord is either increased or unaltered in rats with MIA‐induced OA pain compared with that in saline‐treated controls at the 2 time points studied, providing a putative spinal site of action for the resolvins in this model of OA. It is possible that 17(R)‐HDoHE may still have effects at the level of the joint by activating ALX receptors on synovial cells and possibly reducing release of synovium‐derived nerve sensitization factors such as NGF 43.

We previously demonstrated a significant increase in GFAP immunofluorescence, indicative of astrogliosis and a marker of central sensitization, in the ipsilateral dorsal horn of the spinal cord at later time points in the model of MIA‐induced OA pain 25. In the current study, repeated treatment with 17(R)‐HDoHE from day 14 onward resulted in significant blockade of spinal astrogliosis in the model of MIA‐induced OA pain at the later time point (day 28 after induction of the model), and spinal GFAP expression at this time was correlated with pain behavior. We previously showed that post mortem knee chondropathy scores are significantly and positively correlated with human spinal GFAP mRNA expression 26, confirming the clinical relevance of these spinal markers of central sensitization.

Astrogliosis is associated with numerous models of chronic pain (for review, see ref. 44) and is a proposed switch in the transition from acute to chronic pain mechanisms 45. The ability of 17(R)‐HDoHE to inhibit spinal astrogliosis in preclinical models of OA, along with the clinical associations between joint damage and spinal GFAP expression, supports the need for further investigation of the therapeutic potential of the D series resolvin pathway. The mechanisms by which 17(R)‐HDoHE inhibits astrogliosis may arise as a result of direct effects (although there is little evidence to date) or indirect effects on the spinal signaling pathways that lead to astrogliosis. In particular, activated microglia in the spinal cord play a fundamental role in the development of chronic pain mechanisms and are known to be activated 14–28 days following induction of the MIA model of OA 25, coinciding with the increase in expression of ALX in the ipsilateral spinal cord reported herein.

Microglia are known to express ALX 46, 47, and activation of microglia in models of chronic pain states, including OA, is associated with increased levels of pERK 48, a known spinal target of 17(R)‐HDoHE 21. In addition, the antiinflammatory and pro‐resolution molecule lipoxin A_4_ also signals through ALX 49, and increases in ALX expression seen in the spinal cord in the MIA model may indicate an enhanced antiinflammatory role of lipoxin A_4_.

The results of this series of experiments demonstrate that receptors for both D series and E series resolvins are expressed at multiple sites within the human OA joint, and that the precursor for the D series resolvins reduced OA pain behavior and a key marker of central sensitization (astrocyte activation) associated with chronic pain. These effects, which were not subject to tolerance, at least over a 2‐week period of treatment, likely arise from modulation of both nociceptive input arising from the arthritic joint and modulation of central nociceptive processing. Our findings support the need for further investigation of the therapeutic potential of this new class of analgesics for the treatment of OA pain. Future work could address whether combination treatments that use both 17(R)‐HDoHE and an E series resolvin precursor such as hydroxy‐eicosapentaenoic acid would produce superior analgesic efficacy and potential disease‐modifying properties via the modulation of both resolvin signaling systems.

## AUTHOR CONTRIBUTIONS

All authors were involved in drafting the article or revising it critically for important intellectual content, and all authors approved the final version to be published. Drs. Burston and Chapman had full access to all of the data in the study and take responsibility for the integrity of the data and the accuracy of the data analysis.

### Study conception and design

Huang, Burston, Mapp, Bennett, Ravipati, Pousinis, Barrett, Scammell, Chapman.

### Acquisition of data

Huang, Burston, Li, Ashraf, Mapp, Ravipati.

### Analysis and interpretation of data

Huang, Burston, Li, Ashraf, Bennett, Ravipati, Pousinis, Barrett, Scammell, Chapman.

## Supporting information

Supplementary figure 1. **Expression of the resolvin receptors in the synovium** A: Representative images for DAPI, ED1, ALX and CHEMR23 immunofluorescent staining in the synovium of saline and MIA injected (28 days post injection) rats, scale bar = 35 μm. Space between dotted white lines indicate area used for volocity analysis.
B: Quantification of DAPI positive nuclei in the synovium from Saline or MIA injected rats.
C: Quantification of ED1 positive cells in the synovium from Saline or MIA injected rats.
D: Quantification of ALX positive cells in the synovium from Saline or MIA injected rats.
E: Quantification of CHEMR23 positive cells in the synovium from Saline or MIA injected rats.

Supplementary figure 2. Representative images fro m, ED1, ALX and CHEMR23 immunofluorescent staining in the synovium of saline and MIA injected (28 days post injection) rats when primary antibody was omitted (negative control), scale bar = 30 μm.

Supplementary figure 3. **Effect of systemic administration of 17(R)‐HDoHE on plasma lipid levels in MIA‐treated rats**
Representative extracted LC‐MS/MS ion chromatograms of (A) arachidonic acid, (B) 17R‐HDoHE and (C) resolvin D2 in plasma samples of MIA rats treated with 17R‐HDoHE. Lipids were extracted using Strata‐X polymeric SPE cartridges (200 mg/6 ml).The gradients of solution A‐ 0.02% formic acid in 100% water and solution B‐ 0.02% formic acid in methanol/acetonitrile (1:4, v/v) were used for separation of eicosanoids on ACE C18 (150 × 2.1mm, 3μm) column, chromatograms denote peak height on Y‐axis and retention time on X‐axis. Quantification of arachidonic acid (D), 17–(R) HDoHE (E) and Resolvin D2 (F) in plasma samples harvested at 150 min post 17–(R) HDoHE or vehicle treatment, at day 14 post MIA or saline injection. Data are median and interquartile range (n= 8 rats per group, number of rats in which lipids were detectable per group appear above each bar), comparisons between groups used a Kruskal Wallis test with Dunn's post hoc comparison.

**Supplementary Material**

**Supplementary methods**
