# Supplementary material for: Targeting the D Series Resolvin Receptor System for the Treatment of Osteoarthritis Pain
Source: Arthritis Rheumatol. 2017 Apr 26;69(5):996–1008. doi: 10.1002/art.40001 (PMC5763389; doi:10.1002/art.40001)
Supplement: Supplementary file 1 — Supplementary figure 1. Expression of the resolvin receptors in the synovium A: Representative images for DAPI, ED1, ALX and CHEMR23 immunofluorescent staining in the synovium of saline and MIA injected (28 days post injection) rats, scale bar = 35 μm. Space between dotted white lines indicate area used for volocity analysis. B: Quantification of DAPI positive nuclei in the synovium from Saline or MIA injected rats. C: Quantification of ED1 positive cells in the synovium from Saline or MIA injected rats. D: Quantification of ALX positive cells in the synovium from Saline or MIA injected rats. E: Quantification of CHEMR23 positive cells in the synovium from Saline or MIA injected rats. [file ART-69-996-s001.docx]

Supplementary figure 1. **Expression of the resolvin receptors in the synovium** A: Representative images for DAPI, ED1, ALX and CHEMR23 immunofluorescent staining in the synovium of saline and MIA injected (28 days post injection) rats, scale bar = 35 μm. Space between dotted white lines indicate area used for volocity analysis.

B: Quantification of DAPI positive nuclei in the synovium from Saline or MIA injected rats.

C: Quantification of ED1 positive cells in the synovium from Saline or MIA injected rats.

D: Quantification of ALX positive cells in the synovium from Saline or MIA injected rats.

E: Quantification of CHEMR23 positive cells in the synovium from Saline or MIA injected rats.
