# Supplementary material for: Targeting the D Series Resolvin Receptor System for the Treatment of Osteoarthritis Pain
Source: Arthritis Rheumatol. 2017 Apr 26;69(5):996–1008. doi: 10.1002/art.40001 (PMC5763389; doi:10.1002/art.40001)
Supplement: Supplementary file 2 — Supplementary figure 2. Representative images fro m, ED1, ALX and CHEMR23 immunofluorescent staining in the synovium of saline and MIA injected (28 days post injection) rats when primary antibody was omitted (negative control), scale bar = 30 μm. [file ART-69-996-s002.docx]

Supplementary figure 2. Representative images fro m, ED1, ALX and CHEMR23 immunofluorescent staining in the synovium of saline and MIA injected (28 days post injection) rats when primary antibody was omitted (negative control), scale bar = 30 μm.
