# Supplementary material for: Targeting the D Series Resolvin Receptor System for the Treatment of Osteoarthritis Pain
Source: Arthritis Rheumatol. 2017 Apr 26;69(5):996–1008. doi: 10.1002/art.40001 (PMC5763389; doi:10.1002/art.40001)
Supplement: Supplementary file 3 — Supplementary figure 3. Effect of systemic administration of 17(R)‐HDoHE on plasma lipid levels in MIA‐treated rats Representative extracted LC‐MS/MS ion chromatograms of (A) arachidonic acid, (B) 17R‐HDoHE and (C) resolvin D2 in plasma samples of MIA rats treated with 17R‐HDoHE. Lipids were extracted using Strata‐X polymeric SPE cartridges (200 mg/6 ml).The gradients of solution A‐ 0.02% formic acid in 100% water and solution B‐ 0.02% formic acid in methanol/acetonitrile (1:4, v/v) were used for separation of eicosanoids on ACE C18 (150 × 2.1mm, 3μm) column, chromatograms denote peak height on Y‐axis and retention time on X‐axis. Quantification of arachidonic acid (D), 17–(R) HDoHE (E) and Resolvin D2 (F) in plasma samples harvested at 150 min post 17–(R) HDoHE or vehicle treatment, at day 14 post MIA or saline injection. Data are median and interquartile range (n= 8 rats per group, number of rats in which lipids were detectable per group appear above each bar), comparisons between groups used a Kruskal Wallis test with Dunn's post hoc comparison. [file ART-69-996-s003.docx]

Supplementary figure 3. **Effect of systemic administration of 17(R)-HDoHE on plasma lipid levels in MIA-treated rats**

Representative extracted LC-MS/MS ion chromatograms of (A) arachidonic acid, (B) 17R-HDoHE and (C) resolvin D2 in plasma samples of MIA rats treated with 17R-HDoHE. Lipids were extracted using Strata-X polymeric SPE cartridges (200 mg/6 ml).The gradients of solution A- 0.02% formic acid in 100% water and solution B- 0.02% formic acid in methanol/acetonitrile (1:4, v/v) were used for separation of eicosanoids on ACE C18 (150×2.1mm, 3μm) column, chromatograms denote peak height on Y-axis and retention time on X-axis. Quantification of arachidonic acid (D), 17–(R) HDoHE (E) and Resolvin D2 (F) in plasma samples harvested at 150 min post 17–(R) HDoHE or vehicle treatment, at day 14 post MIA or saline injection. Data are median and interquartile range (n= 8 rats per group, number of rats in which lipids were detectable per group appear above each bar), comparisons between groups used a Kruskal Wallis test with Dunn’s post hoc comparison.
