# Supplementary material for: Targeting the D Series Resolvin Receptor System for the Treatment of Osteoarthritis Pain
Source: Arthritis Rheumatol. 2017 Apr 26;69(5):996–1008. doi: 10.1002/art.40001 (PMC5763389; doi:10.1002/art.40001)
Supplement: Supplementary file 4 — Supplementary Material [file ART-69-996-s004.doc]

**Resolvin D1**

**Resolvin D2**

**17(R)-Resolvin D1**

**17HDoHE**

**Resolvin D1**

**Resolvin D2**

**17(R)-Resolvin D1**

**17HDoHE**
