# Supplementary material for: Targeting the D Series Resolvin Receptor System for the Treatment of Osteoarthritis Pain
Source: Arthritis Rheumatol. 2017 Apr 26;69(5):996–1008. doi: 10.1002/art.40001 (PMC5763389; doi:10.1002/art.40001)
Supplement: Supplementary file 5 — Supplementary methods [file ART-69-996-s005.docx]

**Supplementary methods**

**Animal housing**

Rats were kept on a 12-hr artificial-light/dark cycle (lights on at 7am) in conventional, open-top cages in a holding room kept at a temperature of 22°C and 55% humidity; food and water were available ad libitum.

**OA models**

For the MIA model, rats (160-180g) were anaesthetised (isoflurane (2.5-3%) in 100% oxygen at the flow rate of 1L/min) and received a single intra-articular injection of MIA (1 mg in 50μl of sterile saline per rat; Sigma U.K.) through the infra-patellar ligament of the left knee. Control rats received intra-articular injection of sterile saline (50μl per rat). For the MNX model, rats (180-200g) were anesthetized (isoflurane (2.5-3%) in 100% oxygen at the flow rate of 1L/min), skin over the medial collateral ligament was cut, cautery was used to remove the connective tissue and prevent bleeding, and a section of the medial collateral ligament was removed to expose the meniscus. The meniscus was cut through its full thickness at the narrowest point. The connective tissue layer and skin were closed with coated Vicryl 8-0 and 4-0 sutures, respectively (Ethicon, Livingstone, UK). Sham operated animals underwent an identical procedure with the exception that the meniscus was not transected.

**Pain behaviour expanded**

Weight bearing asymmetry (weight on contralateral limb -weight on ipsilateral limb) was quantified using an Incapacitance Tester (Linton Instrumentation, U.K.). Hindpaw mechanical withdrawal thresholds were determined using von Frey (vF) monofilaments (Linton Instrumentation, bending forces 1–26g) as previously described ([1](#_ENREF_1)). Weight bearing and hindpaw withdrawal thresholds were quantified at baseline and then twice a week thereafter. All behaviour studies were undertaken in a blinded fashion.

**LC-MS/MS quantitative analysis of oxylipins**

The LC-MS/MS method quantified 45 oxylipins and was adapted from a validated method, previously developed by ([2](#_ENREF_2)) and further enhanced by ([3](#_ENREF_3)). Newly introduced analytes were 17-HDoHE, 18-HEPE, 15-deoxy-PGJ2, 6-keto PGF1*α* and 11-dehydro TXB2.

**Equipment:** The HPLC system used was a Shimadzu series 10AD VP LC system (Shimadzu, Columbia, MD, USA). The HPLC Column used was ACE C18 (150 x 2.1 mm, 3 µm I.D) with guard column (Security Guard Cartridges ACE 3 C18 for ID 150 x 2.1 mm column). Mobile phase A was 0.02% formic acid in methanol/acetonitrile (1:4, v/v); mobile phase B was 0.02% formic acid in 100% water. The starting flow rate was 300 µl/min. Strata-X polymeric SPE column (200mg/6 ml) were purchased from Phenomenex, Macclesfield, UK. The evaporator used was a Jouan centrifugal evaporator (Saint-Herblain, France).

MS system used was an Applied Biosystem MDS SCIEX 4000 Q-Trap hybrid triple-quadrupole–linear ion trap mass spectrometer (Applied Biosystem, Foster City, CA, USA) equipped with an electrospray ionisation (ESI) interface.

**Extraction Protocol for Samples:** Samples were stored at -80°C before analysis. Internal standards (10 µl of PGF2a-EA-d4 (2.49 µM), 10 µl of AA-d8 (1 µM), 10 µl of PGD2-d4 (1 µM), 10 µl of 15-HETE-d8 (7.6 µM) were added to each sample or blank sample (0.4 ml water), along with 2 µl of formic acid (98% v/v) and 5 µl of an antioxidant butylhydroxytoluene (BHT). Samples were homogenised in micro centrifuge tubes with the addition of 900 µl of ethanol, followed by a slow vortex stage (10 min) and centrifuged (13000 g, 10 min, 4 °C). The supernatants were transferred to glass tubes and diluted by the addition of 3ml water. The diluted supernatants were loaded to the Strata-X polymeric SPE column (200mg/6 ml, Phenomenex, Macclesfield, UK) that had been preconditioned with 100% ethanol (2ml) and 25% ethanol (4 ml). The SPE cartridge then washed with distilled water (10 ml) and 25% ethanol (5 ml) and was allowed to run it dry. Then the eicosanoids were eluted from the column with ethyl acetate containing 0.0002%BHT (5 ml) and were dried in centrifugal evaporator. The samples were reconstituted in 100% ethanol (100 µl) and transferred to an auto sampler vial prior to LC-MS/MS analysis. The injection volume was 20 µl. It should be noted that our chromatography analysis is able to distinguish between 17R-resolvin D1, resolvin D1 and resolvin D2, but quantification of 17R-resolvin D2 was not conducted (the XICs of the various resolvins can be viewed as a separate word file). Chiral analysis was not undertaken.

**Immunofluorescence**

Study 2 was repeated to explore the effect of 17(R)-HDoHE treatment on MIA-induced spinal astrocyte activation. At the end of the behavioural study (day 28 post MIA injection) rats were overdosed with sodium pentobarbital and transcardially perfused with saline and then 4% paraformaldehyde (Sigma, U.K) at a PH of 7.4. The lumbar spinal cord was removed, post-fixed in 4% paraformaldehyde and stored in 30% sucrose. Spinal cord was then sectioned (40 µm thick sections) and immunohistochemistry conducted using mouse anti-GFAP (1:100, Fisher scientific UK), antibodies. The secondary antibody was Alexafluor 568 conjugated Donkey anti-mouse (1:500) (Fisher scientific). Images were captured and processed identically and any contrast enhancement applied consistently for each image. All images were digitally captured with an 8 bit camera, thus giving grey level (intensity) values of 0–255. GFAP immunostaining was visualised using a 20 × 0.4 NA objective lens on a Leica DMIRE2 fluorescence microscope, running Volocity 5.5 (PerkinElmer) equipped with a Hamamatsu Orca C4642-95 camera. Images were acquired using a typical exposure time of 628 ms. For quantification volocity 5.5 was used to provide the sum of pixels with intensity of between 55 and 254 grey level.

**Knee joint processing**

Tibiofemoral joints were removed and post-fixed in neutral buffered formalin (4% formaldehyde), decalcified in ethylenediaminotetraacetic acid (EDTA) and embedded in paraffin wax. Joints were dehydrated in increasing concentrations of alcohol and then xylene, before being wax embedded. Embedded joints were cut into coronal sections (5μm per section) with a rotary microtome (Slee cut 4060, Slee Medical). Histomorphometry was carried out by an observer blinded to treatment. Haematoxylin and eosin sections were scored for joint morphology. Cartilage surface integrity was scored on 4 coronal sections at 200 um intervals from the anterior half of the knee.

**TRAP staining quantification**

Quantification of TRAP-positive cells (40 X magnification) from one end of the growth plate to the other end was performed using the following criteria; 1) displayed purplish to dark red cytosol, 2) number of nuclei ≥3/osteoclast, 3) located within the subchondral bone area, comprising the area between the cartilage/bone junction and the growth plate. Lightly stained TRAP-positive cells without nuclei identified, or located distal to the subchondral bone were not counted.

**Immunofluorescence of synovial sections.**

From a separate study, saline and MIA injected rats (day 28 post injection), were euthanized by asphyxiation in carbon dioxide. Synovia with patellae from the knee were dissected and snap frozen in optimum cutting temperature compound (OCT) over melting isopentane and then sectioned on a Leica cryostat into 7 μm thick sections. Sections were fixed in 4% paraformaldehyde, and then blocked in PBS containing 5% serum and 0.5% BSA. Immunostaining were performed by incubation the sections for 2 hours at room temperature (RT) with primary antibodies (at a concentration of 1:100) raised against rat ChemR23 (E-19, catalogue number: sc- sc-32652, Santa Cruz), ALX/FPR2 (M-73, catalogue number: sc-66901, Santa Cruz) or CD68/ED1 (catalogue number: MCA341GA, Bio-Rad) respectively, followed by incubation with appropriate secondary antibodies (Alexa flour 488, 1:500) at RT for another 1.5 hours. After extensive washing, sections were counterstained with DAPI for 15 mins, and mounted with Fluoromount mounting medium.

Immuno-stained sections were visualised and areas of the synovium, which included the synovial lining and sublining, was delineated and photographed at 20× by a Leica DMIRE2 fluorescence microscope. Images (TIF files) were acquired using an exposure time of 1800 ms for ChemR23 and ALX or 800 ms for CD68 respectively. For quantification of positive cells, individual TIFF files were exported into Volocity (version 5.0) and the find 2D nuclei/2D spot analysis was used to calculate cell number. Cell counts were collected from 4-5 different fields of view per synovium, and 7-8 different synovium per group were used. Only samples with a clear synovial lining were included in the analyses. Data were then analysed with a two tailed unpaired t-test.

**Polymerase chain reaction**

Briefly, 500 ng of RNA was reverse transcribed with Affinity Script Reverse Transcriptase following the manufacturer’s instructions. cDNA synthesis was conducted at 25°C for 10 min to extend primers, 50°C for 60 min to synthesize cDNA and 70°C for 15 min to terminate the reaction. Synthesized cDNA was pre-amplified for the rat synovia (3 cycles) and clinical samples (8 cycles) because expression levels were lower for some target genes. cDNA samples was amplified using the Applied Biosystem Step OnePlus system (Applied Biosystems, US). The PCR reaction started at 95°C for 20s to activate hot-start Taq DNA polymerase and then 40 cycles at 95°C for 1s to denature and 60°C for 20s to extend the cDNA templates. The expression levels of genes were normalized to β-actin.

**References**

1. Sagar DR, Nwosu L, Walsh DA, & Chapman V (2015) Dissecting the contribution of knee joint NGF to spinal nociceptive sensitization in a model of OA pain in the rat. *Osteoarthritis and cartilage / OARS, Osteoarthritis Research Society*.

2. Zhang JH, Pearson T, Matharoo-Ball B, Ortori CA, Warren AY, Khan R*, et al.* (2007) Quantitative profiling of epoxyeicosatrienoic, hydroxyeicosatetraenoic, and dihydroxyeicosatetraenoic acids in human intrauterine tissues using liquid chromatography/electrospray ionization tandem mass spectrometry. *Anal Biochem* 365(1):40-51.

3. Wong A, Sagar DR, Ortori CA, Kendall DA, Chapman V, & Barrett DA (2014) Simultaneous tissue profiling of eicosanoid and endocannabinoid lipid families in a rat model of osteoarthritis. *J Lipid Res* 55(9):1902-1913.
